# Effect of oxide layers formed by thermal oxidation on mechanical properties and NaCl-induced hot corrosion behavior of TC21 Ti-alloy

**DOI:** 10.1038/s41598-022-23724-6

**Published:** 2022-11-10

**Authors:** Fathy S. Ahmed, Mohamed A. El-Zomor, Magdy S. Abo Ghazala, Ramadan N. Elshaer

**Affiliations:** 1 Tabbin Institute for Metallurgical Studies, Cairo, Egypt; 2 Faculty of Science, Menofia University, Shebeen El-Koom, Egypt

**Keywords:** Engineering, Materials science

## Abstract

In the current study on TC21 Ti-alloy (6.5Al–3Mo–1.9Nb–2.2Sn–2.2Zr–1.5Cr), the thermal oxidation formed oxide layers that considerably influenced mechanical properties (hardness and wear). TC21 specimens were oxidized at 600, 700, 800, and 900 °C for 5, 20, and 50 h. NaCl-induced hot corrosion testing was carried out on raw (un-oxidized) and oxidized specimens at 600 and 800 °C for 50 h. The cyclic testing was performed at 600 °C for durations of 5, 10, 20, 30, 40, and 50 h. The average thickness of the layer grew with increasing oxidation time and temperature. A thin oxide layer (average 0.16 µm) was generated by oxidation at a temperature of 600 °C for a duration of 5 h, and at 800 °C, a large oxide layer of 10.8 µm thickness was formed. The most significant surface hardness of 1000 ± 150 HV_0.05_ was produced for the layer oxidized at 900 °C. On the other hand, the lowest hardness of 360 ± 150 HV_0.05_ was recorded for the raw materials. Best wear resistance had been achieved for specimens oxidized at 800 °C. During NaCl hot corrosion test, the weight loss of the raw specimen was 6.4 mg/cm^2^ due to the flaking off of the corrosion product. However, for specimens oxidized at 600 °C for 50 h, weight loss after corrosion testing was 0.54 mg/cm^2^, less than that of the specimen before corrosion. Oxidized specimens at 800 °C exhibited the best mechanical characteristics and corrosion resistance.

## Introduction

Titanium (Ti) alloys are widely utilized in civil, aviation, and military applications. Among metals, Ti alloys provide various physical and chemical advantages: low density, good mechanical properties, cryogenic temperature resistance, corrosion resistance, and biocompatibility^1–3^. However, these alloys have poor tribological qualities, including a low wear resistance that leads to a limited component lifespan and application range. The surface treatment of Ti alloys has been used to control wear mechanisms and improve tribological properties. An extensive range of surface treatment procedures is accessible such as anodizing, ion implementation, laser shock peening, and thermal oxidation. However, thermal oxidation is the most efficient technique to enhance sliding wear resistance. It may be more efficient in increasing the abrasion resistance of Ti alloys^4–7^.

Thermal oxidation provides high-quality oxide scales primarily dictated by oxidation process parameters. The thermal oxidation technique is simplistic and cost-effective based on the intrinsic attraction of titanium to oxygen and its diffusion at high temperatures. The important parameters of the formed layer, including its thickness, elemental composition, morphology, and tribology, can be modified by choosing a suitable temperature range and time for the thermal oxidation method^8,9^. The oxide layers may have varying thicknesses; they commonly have a rutile oxide (TiO_2_) composition and rough surface structure. The rutile TiO_2_ was formed as a protective layer with continuous, adherent, stable characteristics and excellent corrosion resistance^10^. The disadvantage of this technique is the flaking of the oxide layer at high temperatures. When the thickness of the formed layer increases, it cracks and spalls due to the stresses caused by the difference in thermal expansion between the formed oxide layer and the base metal^11^. Ti alloy has been alloyed with numerous elements (such as Al, Mo, Nb, Zr) to enhance the alloy properties at higher temperatures^12^; notably, their oxidation resistance compared to steels and nickel-based alloys. The composition of Ti alloys has a considerable influence on their corrosion resistance. Elements like Mo, Nb, and Ta increase the corrosion protection of Ti alloys in many corrosive environments, such as systems that induce fluctuations in electrochemical properties ^13,14^.

Studies on Ti alloys showed that they might be subjected to hot corrosion due to the deposition of solid NaCl. During exposure to high temperatures, solid NaCl deposition influences the Ti alloy surface causing the formation of thick, porous, cracked, and non-adherent corrosion layers. Typically, corrosion scales are made mainly of the TiO_2_ rutile and Na-Ti oxides. Oxygen may reach alloys more rapidly by producing substituted volatile chlorides, leading to the splitting of the corrosion product^15,16^.

The current research topics for Ti alloys in aviation include high-temperature oxidation and hot corrosion^17–19^. According to researcher^18^, Ti alloy was exposed to considerable oxidation erosion at high temperature, forming an oxygen-rich layer under the top TiO_2_ layer, inducing oxygen embrittlement and mechanical failure. The Ti_2_AlNb alloy displayed excellent resistance to hot corrosion at 650 °C but was highly detrimental at 750 °C. At 650 °C, the solid salt film slightly interacted with TiO_2_, but at 750 °C, it converted into a molten salt film^19^.

TC21 alloy is a relatively new kind of Ti–Al–Sn–Zr–Mo–Cr–Nb–Si family of dual-phase Ti alloys^20^. This alloy has many advantages: low density, excellent fracture toughness, high strength, and outstanding corrosion resistance. Consequently, TC21 alloy has widespread applications in many industries as structural forgings and bearing parts. It is commonly used for airplane landing gears, connecting components, engine cabin partitions, and engine frameworks with temperature requirements^21,22^. However, due to the low oxidation resistance of this alloy at high temperatures, its use in high-temperature aviation components is still restricted.

Various coating methods, such as electron-beam physical vapor deposition^22^, laser cladding^23,24^, plasma spraying^25^, and plasma electrolytic oxidation (PEO)^26^ are used, aiming to enhance the high-temperature oxidation resistance and mechanical properties. Other methods of hot dipping^27^ and electron beam melting^28^ have also been studied. These methods require complex and expensive equipment to apply; some are unsuitable for parts with complex shapes. The most recent research on the TC21 alloy focuses on its mechanical characteristics and microstructure change following heat treatment^20,29^. However, a lesser number of researches were reported on the influence of oxide layers created by the thermal oxidation as an easy, suitable for parts with complex shapes, and less costly method for improving the mechanical characteristics and the hot corrosion behavior of TC21 Ti-alloy. Therefore, this research seeks to evaluate the influence of oxide layers formed by the thermal oxidation at 600, 700, 800, and 900 °C for 5, 20, and 50 h on mechanical characteristics (micro-hardness and wear) of TC21 Ti-alloy. In addition, the effect of oxidation of TC21 alloy at 600 and 800 °C for 50 h on NaCl-induced hot corrosion behavior at 600 °C was examined and compared with the raw material.

## Material and experimental procedures

### Material

**Figure 1:**
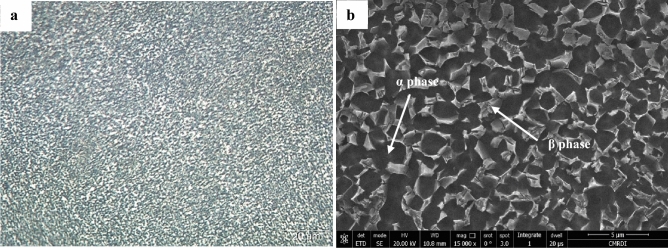
As received α/β equiaxed microstructure of TC21 Ti-alloy (**a**) Optical micrograph (**b**) SEM.

TC21 Ti-alloy bars (7 mm diameter and 140 mm length) employed in this experiment are Ti-based alloy, including 6.5Al–3Mo–1.9Nb–2.2Sn–2.2Zr–1.5Cr–0.09Si (wt.%). The density of TC21 alloy was 4.63 g/cm^3^. The alloying element as Mo, Nb, and Cr are β stabilizers and used for improving hardenability and oxidation resistance. Al is an α stabilizer and used for increasing tensile strength, creep strength, and elastic moduli. Zr is a neutral element and used for increasing strength at low and intermediate temperatures. Solid solutions of Sn are often strengthened with aluminum, due to its extensive solubility in both α and β phases. Also added to achieve a good combination of strength and temperature capability when added in the range of 2–6 wt. %. This alloy was manufactured by Baoji Hans Material Technology Co. Ltd. in China. As-received alloy was forged and annealed in the α + β phase region to achieve the equiaxed microstructure, as seen in Fig. 1.

### Experimental procedures

As-received specimens were solution treated at 990 °C with a heating rate of 10 °C/min and holding time of 15 min to achieve equilibrium, followed by a furnace cooled to 810 °C with a cooling rate of 1 °C/min and holding time of 20 min. Then, specimens are cooled down to room temperature by using water quenching (WQ). Consequently, aging treatment was applied at 600 °C for 6 h and finally air-cooled to room temperature. For the thermal oxidation procedure, specimens were shaped at 7 mm in diameter and 12 mm in length. Before being exposed to the thermal oxidation process, the specimens were ground using different SiC papers (grit size 200 up to 800). Finally, the specimens were ultrasonic cleaning clean **in** ethanol for 10 min and then air-dried at ambient temperature. The thermal oxidation mothed of TC21-Ti alloy was heated in a Carbolite EVC compact vertical tube furnace, United Kingdom. Specimens were heated at 600, 700, 800, and 900 °C for varying 5, 20, and 50 h, then air cooling. The weight increase per surface area (mg/cm^2^) for each oxidation specimen was determined by dividing weight variation by specimen (A) area.

Scanning electron microscopy (SEM), FEI INSPECT 50S, and adding Energy Dispersive Spectroscopy (EDS), Bruker AXS-Flash Detector 410-M, were applied to research surfaces and cross-sections, and distribution of elements in the layer of oxidized materials. Specimens were produced using standard metallography procedures and etched using a Kroll solution of 3% HF, 30% HNO_3_, and 67% H_2_O. Phase compositions of raw, oxidized, and corroded specimens were determined using X-Ray diffraction (XRD), PANalytical X'PERT PRO, with a diffractometer using Cu K α1 radiation with a 2θ range of 20°–80° and recording data at 0.02 degrees with steps at a speed of 0.004 degree/min.

The microhardness of raw and oxidized specimens was measured using Vickers LECO LM700 microhardness tester with an applied load of 50-gf for 15 s. Also, micro-hardness was employed to investigate the oxidized specimens from surface to core. Five indentations were applied to each specimen, and the average was recorded. Testing dry sliding wear at room temperature was performed with pin-on-ring tribometer model TNO TRIBOMETER (Netherlands). Wear specimens with cylindrical pin form of 7 × 12 mm were fastened against an abrasion revolving stainless steel ring) hardness is 63 HRC( at a speed of 1.5 m/s and load of 40 N for 20 min. Three specimens of each condition were submitted to wear testing and the average was reported. The test results were assessed depending on the specimen's weight reduction.

The raw and oxidized (600 and 800 °C for 50 h) specimens were salt corrosion tested at 600 °C for 5, 10, 20, 30, 40, and 50 h, then air cooling. This salt spray corrosion test simulated such corrosive conditions in the marine atmosphere. In numerous stages, spraying used an aqueous solution of NaCl with a concentration of 3.5 mg/cm^2^. The deposition process established in the laboratory consists of heating the specimen surfaces to a temperature of roughly 65–75 °C and spraying them with a supersaturated solution of NaCl. As the sprays advance, water evaporates, enabling the salt to crystallize on the specimen surfaces. The salt steam was sprayed on and off repeatedly. A fresh layer is applied when the NaCl solution reaches 3.5 mg/cm^2^ on the specimen surfaces. Corrosion testing was applied by heating in a Nabertherm muffle furnace with a regulated environment (maximum temperature of 1200 °C) in the presence of a salt deposit NaCl. The specimens were weighted to an accuracy of 0.1 mg by the Mettler Toledo scale before and after oxidation, wear, and corrosion testing.

## Results and discussion

### Microstructure of heat-treated specimen

**Figure 2:**
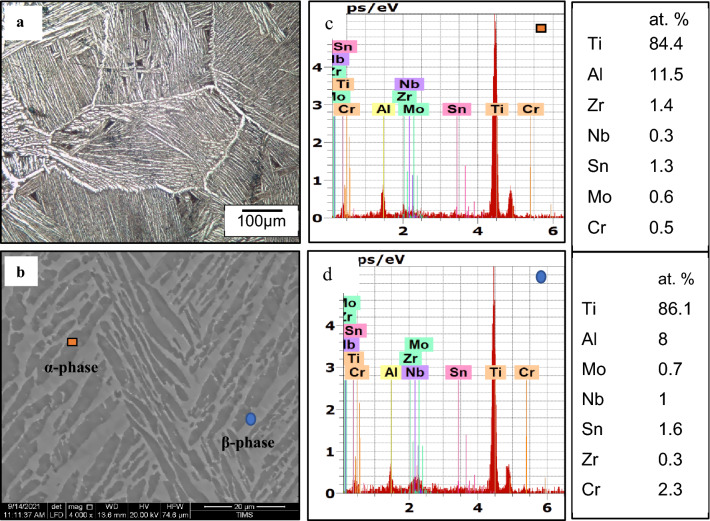
Lamellar microstructure of TC21 Ti-alloy: (**a**) OM (**b**) SEM and (**c**) EDS analysis for α-phase; (**d**) for β-phase.

The microstructure consists of a lamellar α (HCP) and β (BCC) phases, as shown in Fig. 2a. The lamellar α-phase has a random orientation with a length of 20–50 µm and a width of 3–4 µm, as displayed in Fig. 2b. EDS technique was used to analyze both α and β-phases as shown in Fig. 2c,d. The α-stabilizing elements such as Al was found in high content in the α-phase, and some β-stabilizing elements (Nb and Cr) were identified in low content, Fig. 2c. In the β-phase, on the other hand, a high amount of β-stabilizing elements (such as Nb = 1% Mo = 0.7% and Cr = 2.3% at.) was found, as shown in Fig. 2d.

### Thermal oxidation kinetics 

**Figure 3:**
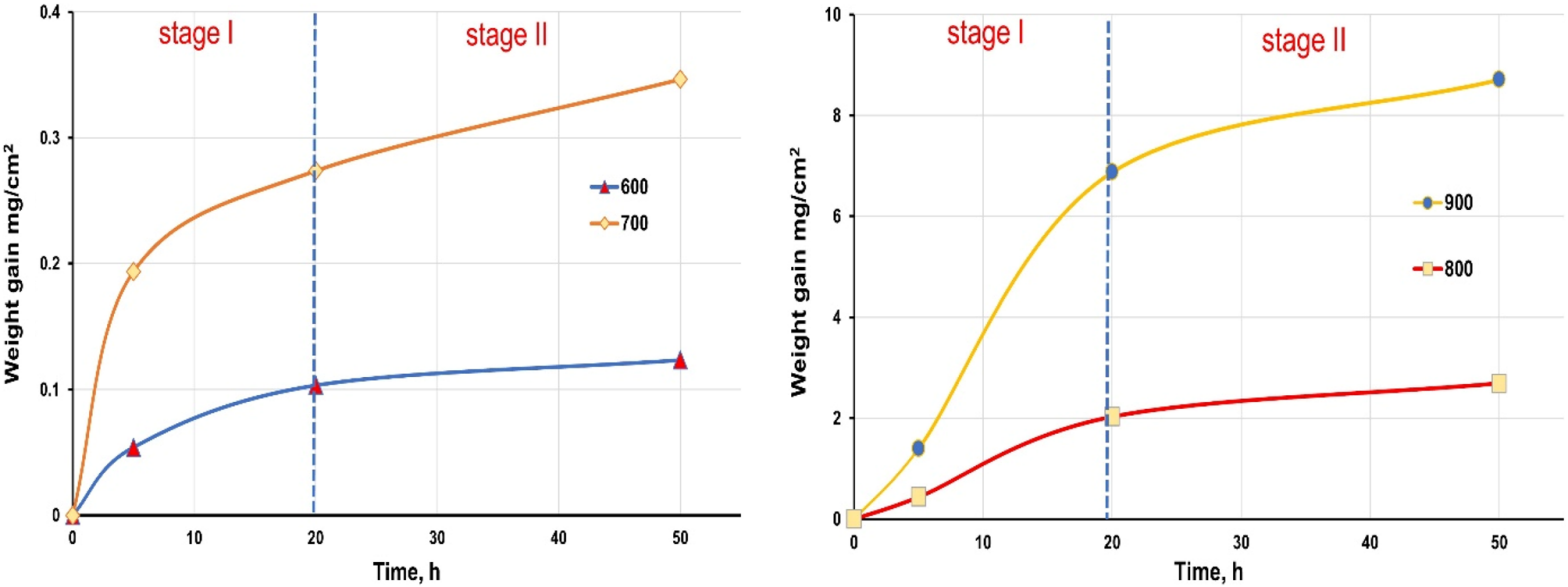
Thermal oxidation kinetics of TC21 Ti-alloy as a function of oxidation time at different temperatures.

Oxidation kinetics are displayed in Fig. 3 as the parabolic curves of weight gain per surface area (ΔW/A) vs. the time and temperature in the thermal oxidation process. This method involves the adsorption of oxygen molecules from the air, oxides nucleation, production of a thin oxide layer, and subsequent growth to a thicker scale^30^. At 600 and 700 °C, oxidation was compatible with the parabolic oxidation law, according to the reference data^31,32^. The oxidation process rises with increasing temperature from 600 to 900 °C. The most significant growth in the specimen weight is observed at 900 °C.

The weight gain due to oxidation of TC21 alloy was found to be more affected by temperature than by time of oxidation, as indicated in Fig. 3. Initially, the weight gain rapidly increased with increasing duration up to 20 h and ultimately demonstrated a steady-state up to 50 h in cases of 800 and 900 °C. As indicated in Fig. 3, this procedure may be split into two steps. In stage I, there was a high rate of weight gain. Then the weight gain approached saturation or semi-steady-state compared to stage I, denoted as stage II, where the slowdown of increase in weight gain is due to the formation and growth of oxide layer and the reduction of O diffusion through it.

The oxidized specimen at 600 °C displayed the lowest weight gain, 0.12 mg/cm^2^. In contrast, the oxidized specimen at 900 °C exhibited the highest weight gain, 8.71 mg/cm^2^. The weight gains of the oxidized specimen at 900 °C are roughly 70, 25, and 3 times higher than that of the oxidized specimens at 600, 700, and 800 °C, respectively. This result is consistent with McReynolds and Tamirisakandala's results under comparable exposure settings^33^; nevertheless, there was variance in the Ti alloy composition. Kumar et al.^34^ studied the effect of thermal oxidation on the surface of commercially pure (CP) titanium and reported that no change in weight of the oxidized specimen could be observed below 600 °C. Hence, the suitable temperature range for thermal oxidation should be 600–800 °C. TC21 Ti-alloy subjected to laboratory air oxidation has a lower weight gain compared with Ti–6Al–4 V, which is attributed to the positive effect of Mo, Nb, and Si alloying constituents on the oxidation behavior of Ti alloys^6^.

**Figure 4:**
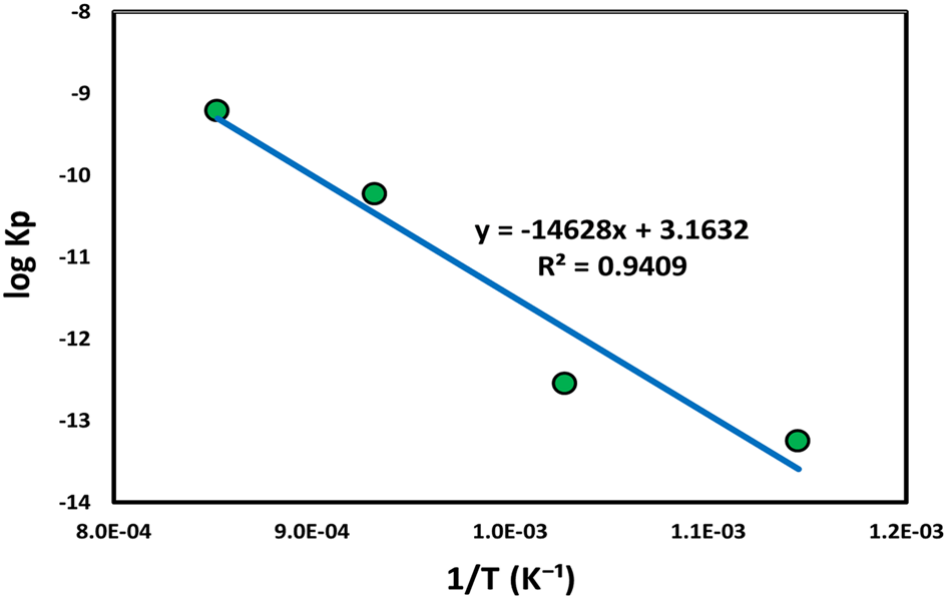
Arrhenius equation represents parabolic constant (log K_p_) with temperature (1/T) for thermal oxidation at 600–900 °C for 50 h.

Based on the findings (Fig. 3), the temperature had a major effect on the intensity of the oxide layer produced on Ti alloy. In addition, thermal oxidation time was only of minor relevance. Anioek's previous study supports this conclusion^6,34^. The following parabolic constant values of oxidation rate K_p_ of TC21 alloy are recorded in Table 1. According to the obtained data, it was found that the constant K_p_ of TC21 alloy increases as the oxidation temperature rises. The Arrhenius energy equation was constructed employing activation energy from the thermal oxidation method by relating K_p_ to T (temperature)^31^.

1 $$ {\text{K}}_{{\text{p}}} = {\text{ K}}_{{\text{o}}} {\text{exp}}^{{ - \, ({\text{Q}}/{\text{RT}})}} $$

where K_o_ represents the pre-exponential factor, Q signifies activation energy, R (8.3143 J/(mol K)) is a gas constant, and T (°K) is temperature. The value of Q may be approximated by plotting log K_p_ with 1/T, as illustrated in Fig. 4. The most suited line to the slope is equal to Q/2.303R. The activation energy (Q_ox_) of TC21 Ti-alloy laboratory air oxidation for 50 h is assessed at 280 kJ.mol^-1^. Guleryuz et al.^31^ examined the oxidation dynamics of Ti–6Al–4 V alloy. They observed activation energies of oxidation between 600 and 700 °C were 276 and 191 kJ/mol^-1^, respectively, for an oxidation duration of 72 h. Kumar et al.^34^ studied the oxidation kinetics of CP Ti alloy and observed 275 kJ/mol^-1^ activation energy at oxidation temperatures between 500–800 °C (up to 72 h exposure period). Also, Aniołek^35^ observed 278 kJ/mol^-1^ activation energy of CP titanium alloy at oxidation temperatures between 600–800 °C for 72 h oxidation duration. However, 170 kJ/mol^-1^ activation energy for Ti–6Al–7Nb alloy was found at the same values. The activation energy of 322 kJ/mol^-1^ was reported for TC21 alloy^36,37^.

**Table 1:** Parabolic constant values of oxidation rate K_p_ at various temperature.

Temperature, °C	600	700	800	900
K_p_ (g^2^cm^-4^ s^-1^)	5.6 × 10^–14^	2.8 × 10^–13^	5.9 × 10^–11^	6.2 × 10^–10^

### Analysis of oxidized specimens

**Figure 5:**
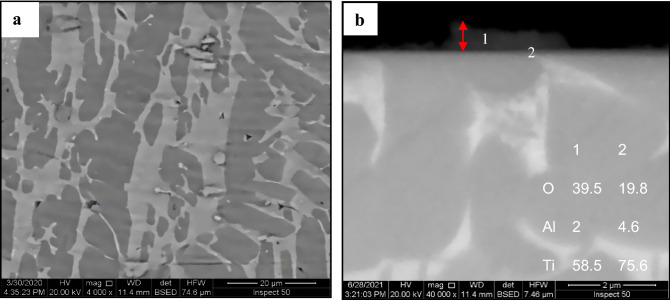
SEM and EDS analysis for oxide specimens at 600 °C after 50 h exposure (**a**) morphology, and **(b)** cross-section.

**Figure 6:**
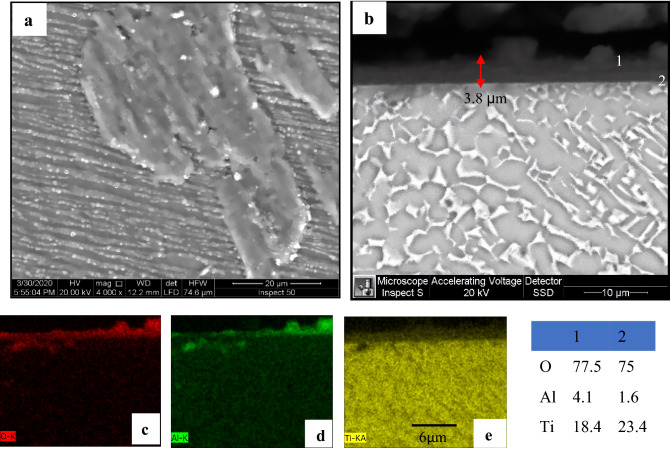
SEM and EDS analysis for oxide specimens at 700 °C after 50 h exposure presents (**a)** morphology, (**b**) cross-section, and (**c, d, e**) element map concentration.

**Figure 7:**
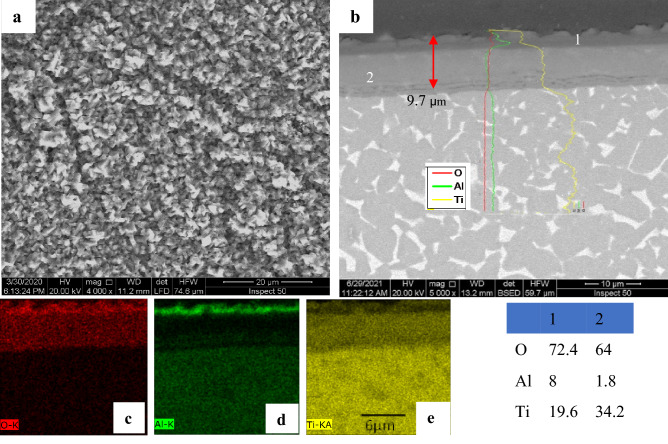
SEM and EDS analysis for oxide specimens at 800 °C presents (**a**) surface morphology, (**b**) cross-section, and (**c–e**) element concentration map.

**Figure 8:**
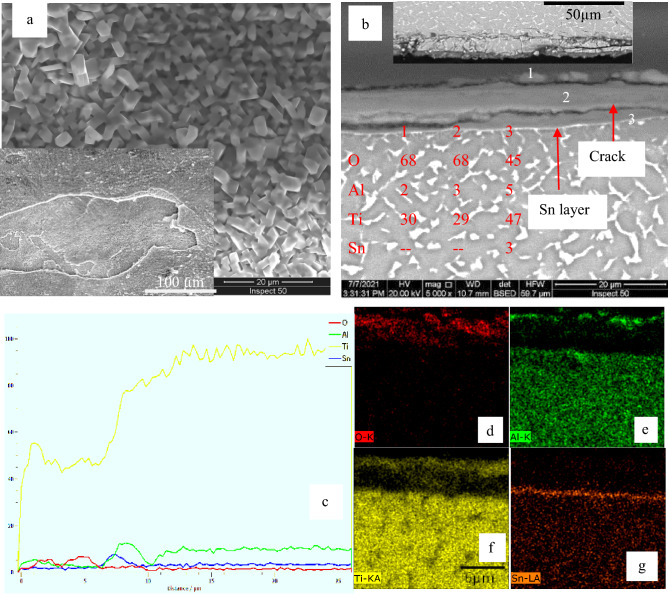
SEM and EDS analysis for oxide specimens at 900 °C (**a**) surface morphology, (**b**) cross-section, (**c**) line element distribution and (**d–g**) element concentration map.

Visual inspections of oxidized materials display a unique coloration and adhering surface condition. Increasing temperatures from 600 to 700, 800, and 900 °C resulted in brown, dark brown, and blackish-green in oxidized specimens. Change in color of TC21 alloy following thermal oxidation is correlated to the growth in thickness of the layer and the accompanying change in the interference of incoming light radiation. Differing the thickness and composition of the covering layer may explain the color variances^36^. SEM micrographs and EDS analysis in Figs. 5–8 illustrate the surface morphology, cross-sections microstructure, and composition of components of oxide layers generated at temperatures from 600 to 900 °C for 50 h. Examination of the surface of specimens at thermal oxidation temperature 600–900 °C demonstrated a varied morphology for each oxidation temperature. Specimens oxidized below 800 °C exhibited a smooth surface comprised of the microstructure and batches of thin oxide grains. The specimens oxidized at temperatures 800 and 900 °C have a rough surface of oxide grains. The surface roughness increases with increasing temperature and thermal oxidation duration^6,38^.

A homogenous microstructure was seen at the surface for oxidized specimens at 600 and 700 °C, as depicted in Figs. 5a and 6a. The oxide layer formed at 600 °C seemed adherent, continuous but with inhomogeneous thickness. The EDS analysis of the oxide layer formed at 600 °C was composed of high O concentration at (point 1) and lower O concentration at oxide/metal interface (point 2), as seen in Fig. 5b. Larger areas of oxide layer aggregations have begun to appear adherent, continuous and with homogeneous thickness at 700 °C, although areas of the base microstructure still observed. Hence, the oxide layer consisted of the same O concentration at points 1 and 2, as illustrated in Fig. 6b.

The morphology of oxidation layers at 800 and 900 °C radically differs from that at lower oxidation temperatures. SEM micrograph of Figs. 6b and 7b revealed continuous and well-adhered oxide layers produced during oxidation at 700 and 800 °C with no pores or cracks forming in the cross-section. Tiny oxide grains were observed in the layer produced at 800 °C, as shown in Fig. 7a. SEM, EDS analysis, line scan, and element concentration map at 800 °C are shown in Fig. 7, which indicates that the oxide layer had high O concentration at point 1 and 2. The top surface layer found a high Al concentration (point 1). EDS map, Fig. 7d, depicts a regular and continuous Al layer with was 2.1 μm thickness.

After oxidation at 900 °C, a thick and porous oxide layer with limited adherence was formed, as shown in Fig. 8. The oxidation layer at 900 °C has a rough surface with large oxide grains, as shown in Fig. 8a. Cracked and flaking off oxide layers were produced on the surface as shown in different steps illustrated in Fig. 8b. EDS analysis of the oxide layer indicated the same concentration O and the same concentration Ti at point 1 on the top surface and point 2 on the middle of layer, as demonstrated in Fig. 8. The line scan, and element concentration maps of the oxide layer indicated Al-rich irregular layer having a thickness of 1.4 μm; as shown in Fig. 8e. Also, Sn layer having a thickness of 0.5 μm was observed at the interface of the oxide layer and base metal. A large variation was found in the thickness of the oxide layer at 900 °C, where the oxide layer had flaked off from the base metal. Figures 5–8 indicate that EDS analysis showed nearly the same O concentrations in oxide layers for oxidation temperature at 700, 800, and 900 °C. Al layer was measured at the surface of the oxide specimens at 700, 800, and 900 °C but not observed at 600 °C. SEM cross-section images of oxide layers at temperatures fluctuating from 600 to 900 °C with 100 °C step are presented in Figures 5–8b.

The spallation and high surface roughness are restricted by changes in the thermal expansion coefficient between the layer and base metal^31,39^. Because TC21 alloy comprises various alloying elements, the active oxidation loop leads to a stratification of oxide scale at increased temperatures. The flaking-off oxide layer was shown at oxidation 900 °C in the case of TC21. The spallation and oxide grains size were observed after oxidation of Ti alloy at different temperatures and duration depending on its chemical composition^40,41^.

**Figure 9:**
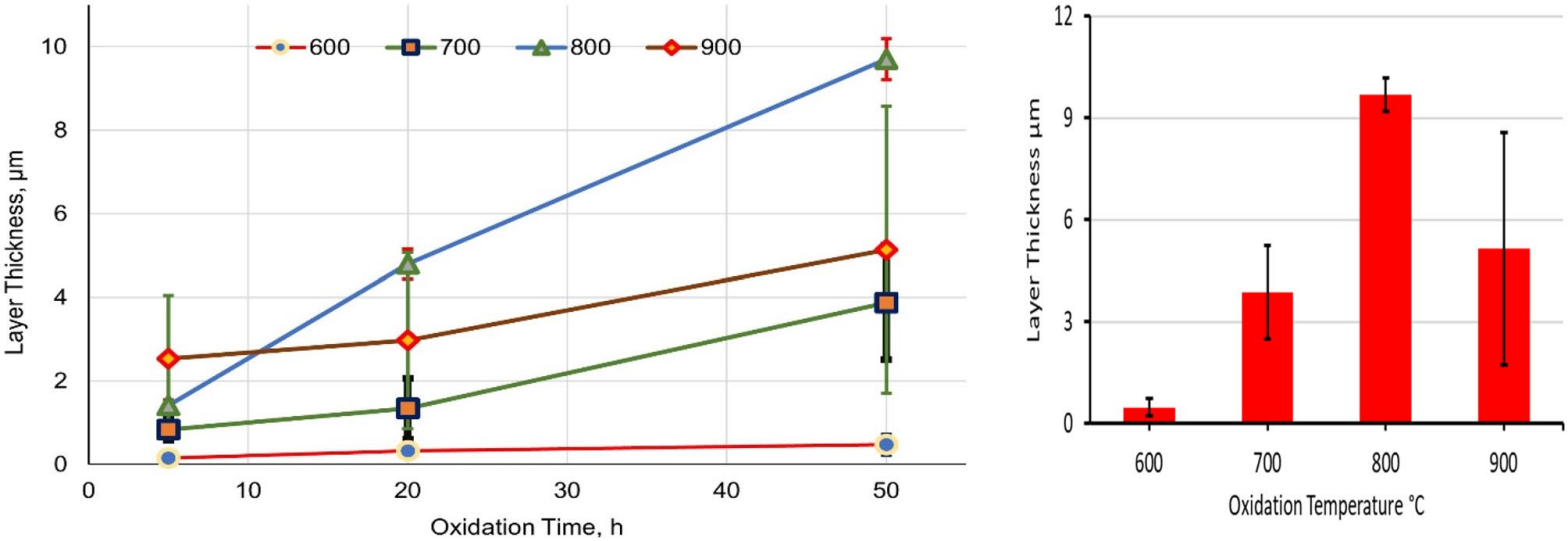
Average thickness of oxide layers after oxidation at different temperatures and times.

Figure 9 demonstrates the connection between average layer thickness and oxidation time at different temperatures. The thickness oxide layer was proportional to oxidation temperature and duration, but at 900 °C, a high error measuring read. As a result of a difficult-to-view thin layer of oxidation at 600 °C, measurement accuracy was low. The effect of temperature represented in oxide layer thickness at 700 °C increased by approximately eightfold compared to 600 °C. The thickness oxide layer at 700 °C for 50 h was (3.8 ± 0.3 µm) compared to the layer at 600 °C for 50 h (0.47 ± 0.25 µm). Suitable thickness was measured at 700 and 800 °C, where the layer was homogenous, continuous, and covered the surface without spallation. The thickness oxide layer at 800 °C was nearly linearly proportional to oxidation time. The average oxide layer thickness was 9.7 ± 0.5 and 5.1 ± 3.4 μm, respectively, after oxidation at 800 and 900 °C, where a high error bar for oxide at 900 °C resulted in the spallation of the oxide layer.

The oxidation procedure at 800 °C permitted a good layer covering the investigated area. The oxygen concentration decreased gradually from interface oxide metal, reaching the same level in base metal. In the oxidation of Ti-6Al-4 V alloy, the layers formed offered a larger thickness than that of TC21 alloy at the same temperature since TC21 alloy comprises alloy components that strongly impact the oxidation process^4^.

**Figure 10:**
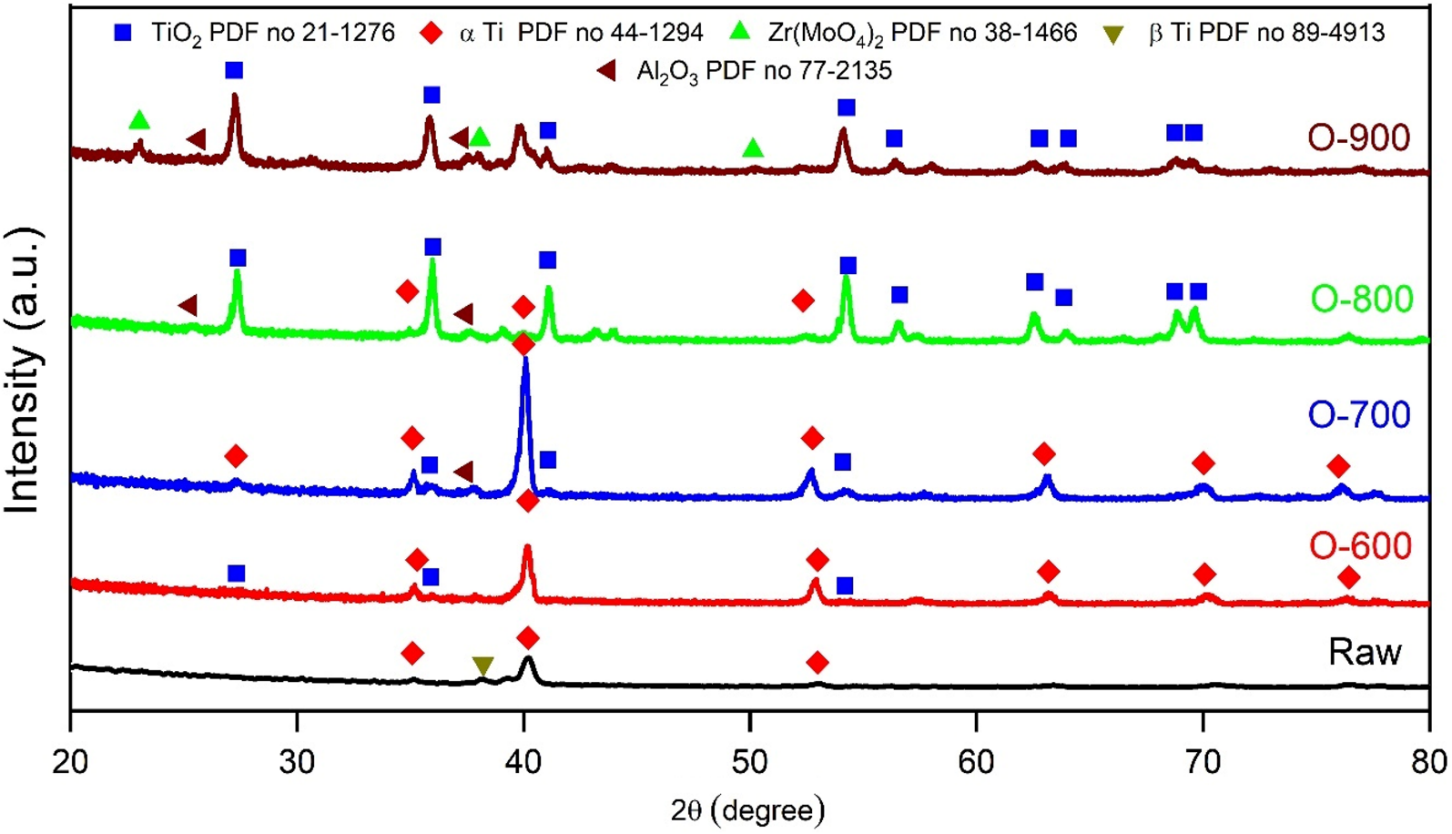
XRD of raw and oxidized specimens at a temperature of 600, 700, 800, and 900 °C for 50 h.

XRD patterns of raw specimens and the formed oxide layers of specimens oxidized at temperatures of 600, 700, 800, and 900 °C for 50 h are shown in Fig. 10 and summarized in Table 2. The raw specimen comprises α-Ti and β-Ti phases (with PDF numbers 44-1294 and 89-4913, respectively). The α-Ti peaks are prominent, and Ti_2_O (with PDF number 21-1276) phases are relatively minor when specimens are oxidized at 600 °C. Peaks of the rutile phase will occur as oxidization temperature rises, corresponding fall in the intensity of α-Ti and other oxide peaks. The α-Ti phase of specimens oxidized under 800 °C is visible and very small at 900 °C to disappear. As shown from the oxidized pattern at 800 and 900 °C, the predominant component of the oxidation layer remains TiO_2_.

**Table 2:** Results of the phase composition examination of TC21 Ti-alloy after thermal oxidation.

	Raw	Oxidized temperature, °C			
		600	700	800	900
Phases	α-Ti β-Ti	α-Ti Ti_2_O	α-Ti TiO_2_ Al_2_O_3_	α-Ti TiO2 Al2O3	TiO2 Zr (MoO_4_)_2_ Al2O3

Most researches show rutile TiO_2_ is the major phase in the thermal oxidation of titanium. The rutile TiO_2_ is more predominant at rises oxidation temperatures. The rutile phase has more corrosion resistance than the anatase phase^6,42^. XRD analysis at thermal oxidation indicates the Al_2_O_3_ phase (with PDF number 77-2135) has appeared, where conformed with observed as a thin layer detected by element concentration map at 700, 800, and 900 °C. An appearance of the Zr(MoO_4_)_2_ (with PDF number 38-1466) phase at high temperatures of 900 °C. The TiO_2_ and other oxides compose most of the oxide layer due to the high solubility of oxygen in titanium and its oxides^19^.

### Hardness evaluation

**Figure 11:**
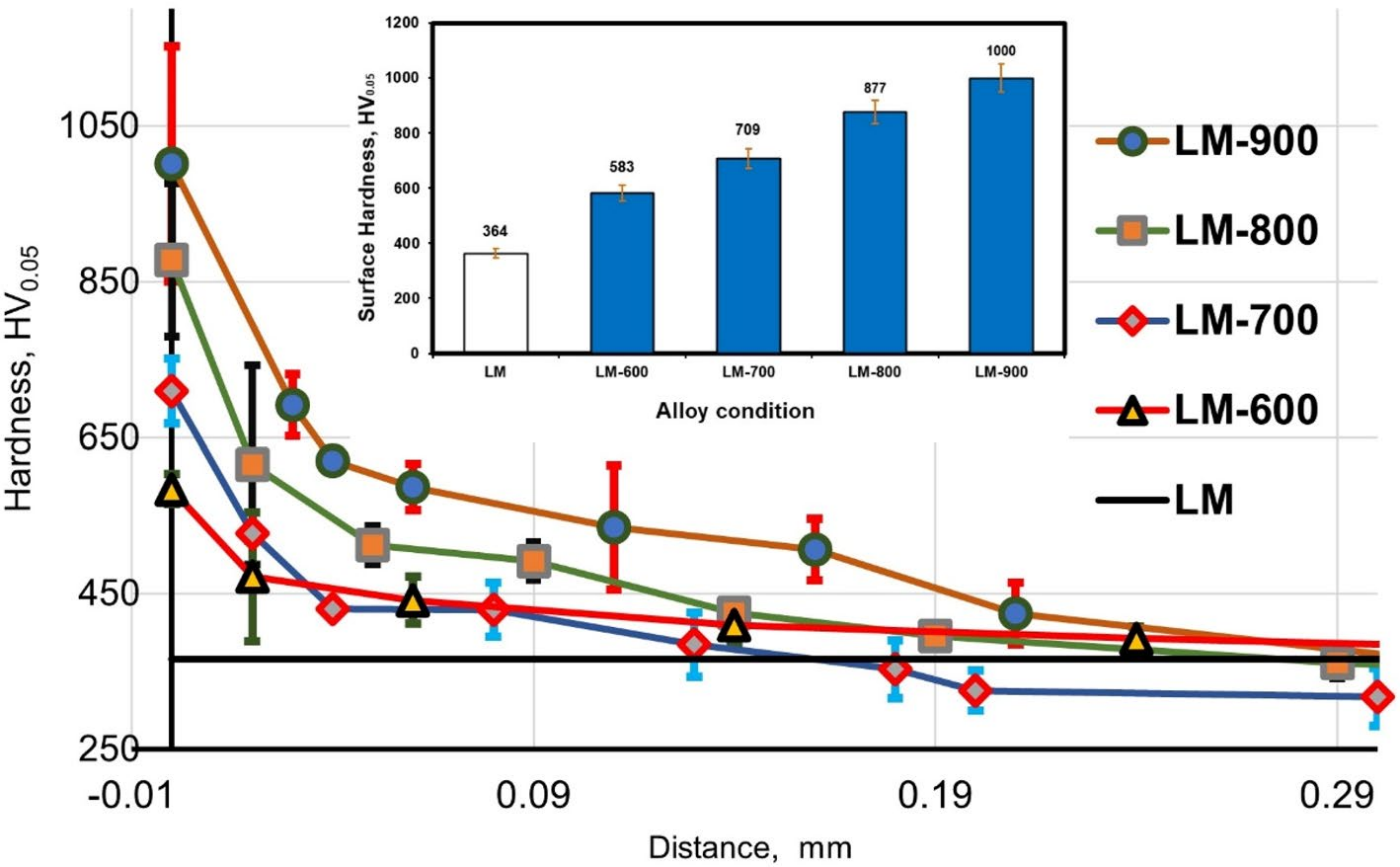
Variation of surface and depth hardness with oxidation parameters for raw and oxidized specimens.

The influence of oxygen on Ti alloys significantly increases hardness, according to several studies^31,42^. Oxidized specimens’ hardness depends on the oxidation temperature and depth of the oxidation, as illustrated in Fig. 11. The TC21 alloy substrate has a micro-hardness of 364 ± 15 HV_0.05_. Specimen oxidized to 900 °C exhibited the maximum surface hardness, reaching 1000 ± 150 HV_0.05_. Oxidized surfaces heated to 600 and 700 °C demonstrated a hardness increase by two times that of raw surfaces. Three times as hard as the raw surfaces were the oxidized specimens at 800 and 900 °C. Increase the hardness of the oxidized surface as the alloy hard oxide layers increase^30^. The hardness value has been lowered steadily as the distance from the oxide contact substrate grows to the average alloy value. Ti alloys let oxygen be dissolved beneath the scale in a substrate, which results in a rise in hardness in the under-oxide layer^4^. The dissolved oxygen atoms in the lattice distortion let to increase in c/a ratio so that hardness increases after thermal oxidation treatment^32^. The oxide layer was mainly formed of the TiO_2_ rutile phase with the properties of high hardness and good thermal stability^43^. According to Guleryuz and Cimenoglu^44^, microhardness values of thermal oxidation of Ti alloys increased with increasing oxidation temperature and time.

### Wear properties

**Figure 12:**
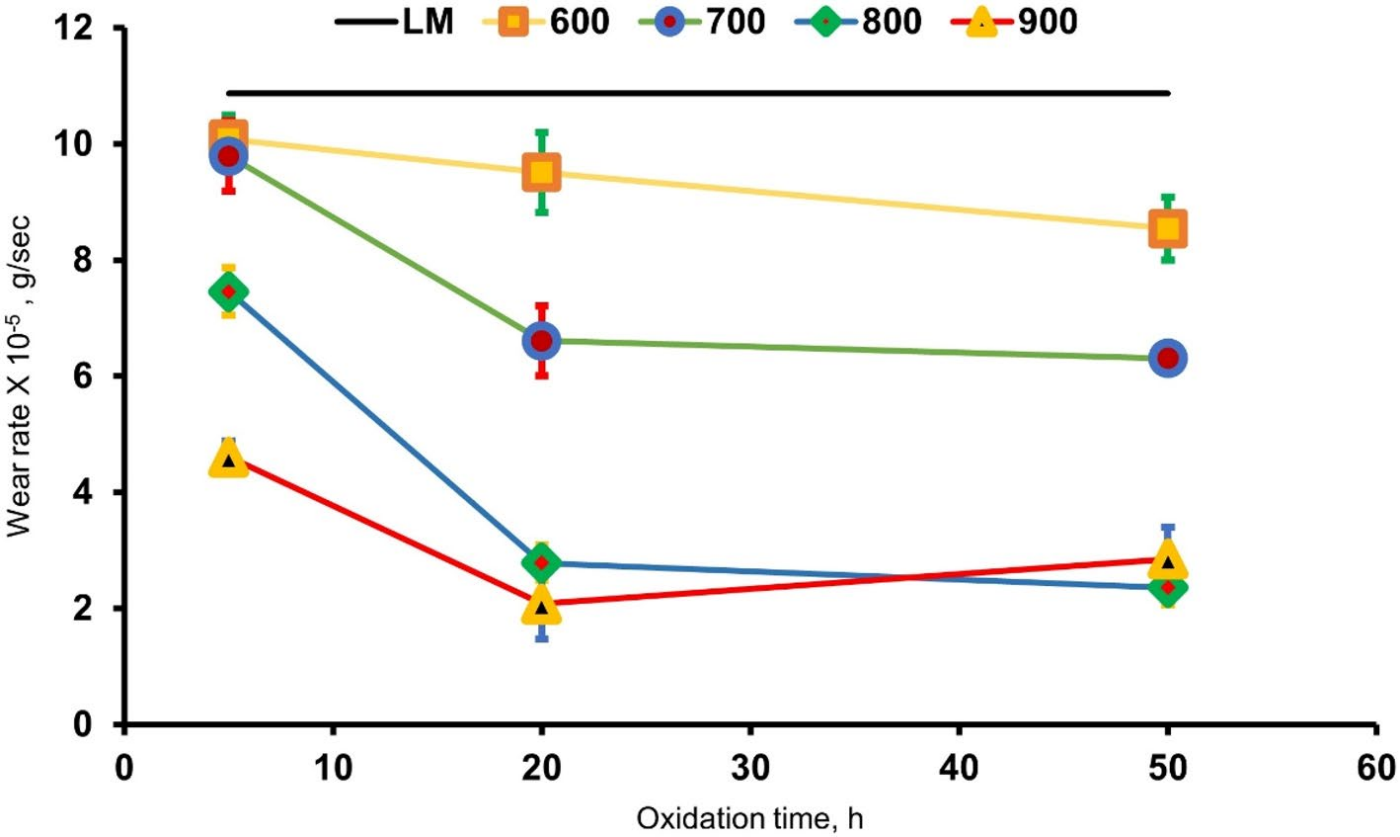
Wear rate of raw and oxidized specimens at different temperatures and time.

The wear test values generated for raw and oxidized specimens at varied temperatures and periods are presented in Fig. 12. The wear rate of raw specimens was 10.9 × 10^–5^ g/sec, the highest value compared to oxide specimens. The wear rate of the oxidized specimens depends on temperature and duration of oxidation, where oxidation time and temperature increase result in a drop-in wear rate. However, temperature is more effective than time. The wear rate of oxidation at 600 °C for 50 h specimens was 8.5 × 10^–5^ g/sec, a limited influence because of the very thin oxide layer formed. The increased oxidation to 700 °C for 50 h specimens wear rate was 6.3 × 10^–5^ g/sec, where the wear rate improved by 21 and 36%, for oxidized at 600 and 700 °C for 50 h specimens, respectively, compared with raw specimens. SEM indicated the oxide layer's good properties as continuity and adhesion, with a high degree of oxidation on the surface. The best result of wear rate was obtained for oxidized specimens at 800 and 900 °C. The wear rate of oxidation at 800 and 900 °C for 50 h specimens were 2.3 × 10^–5^ and 2.8 × 10^–5^ g/sec, respectively, representing an improvement of 78 and 73% compared to raw specimens. Improved wear characteristics are attributed to limiting deformation in the top oxide layer and the lowering of shear strength^8^. According to the Archard rule, wear rate is inversely proportional to material hardness, which indicates that the rise of material hardness increases wear resistance^11^. The surface morphologies, composition, and hardness of the oxides formed at 900 °C led to spallation of the oxide layer and reduced the layer adhesion, affecting the wear rate. X-ray analysis of oxidized specimens indicated the formation of the rutile TiO_2_ phase, which has a hardness of 9.8 GPa, and other hard oxide phases with different compositions for different oxidation temperatures. The hard rutile TiO_2_ has the best properties, where abrasive wear is the dominating wear mechanism, leading to a reduction of wear rate by fourfold in the present study. This result agrees with other work, which reported a 4 – sixfold improvement in abrasive wear resistance^8^.

### NaCl hot corrosion

**Figure 13:**
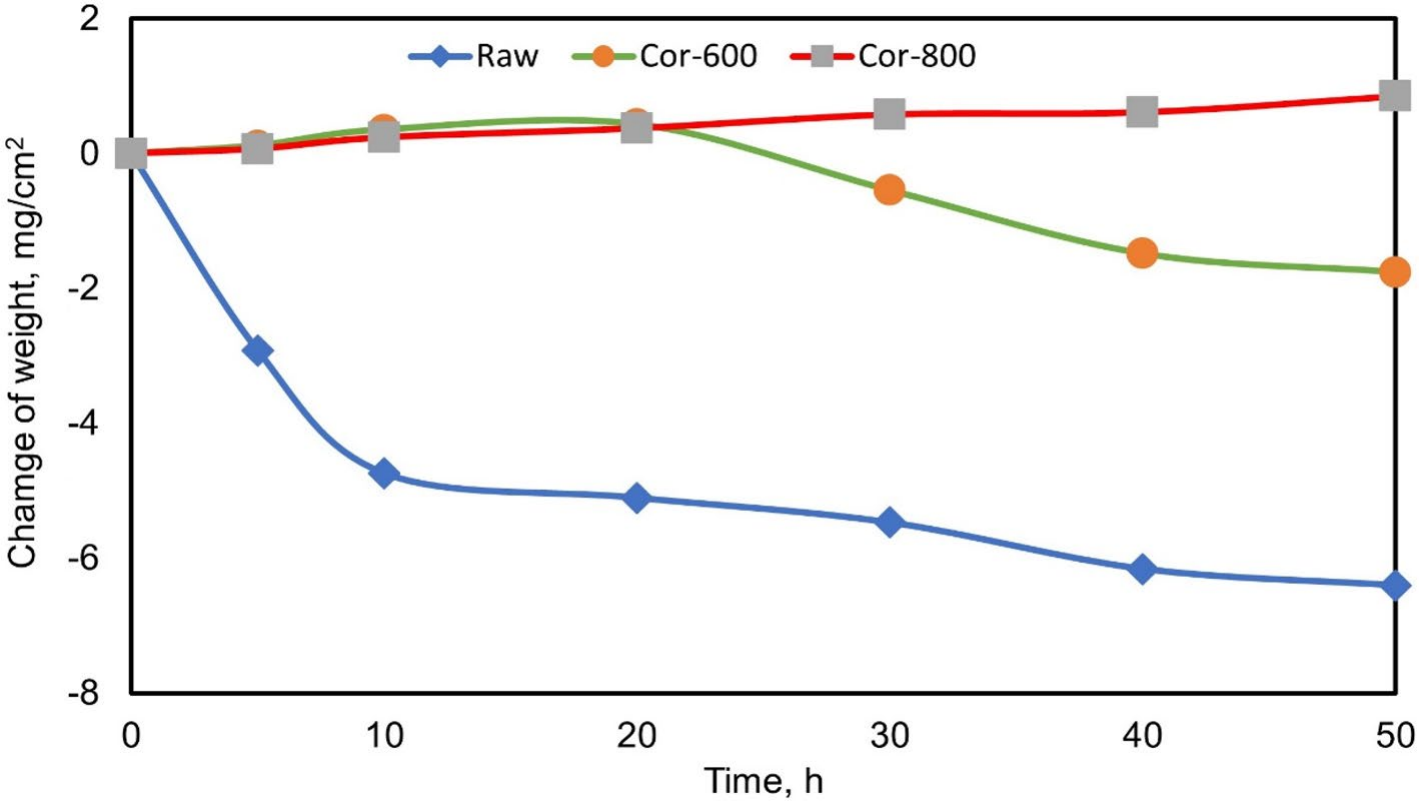
Variation of the weight of raw and oxidized (600 and 800 °C for 50 h) specimens after hot corrosion at 600 °C for 5, 10, 20, 30, 40, and 50 h.

Figure 13 illustrates the weight fluctuation of raw and oxidized (600 and 800 °C for 50 h) specimens following the hot corrosion cycle at 600 °C for 50 h with a 10 h step. The presence of solid NaCl hot corrosion deposits on the TC21 alloy surface has a highly damaging impact. For raw specimens, the appearance of metallic-white color was changed to black after corrosion. A harmful effect in the material is measured as 6.4 mg/cm^2^ weight loss compared to the initial weight of metal because of corrosion scale spalling. The weight of loss dropped after the first 10 h, but the loss rate between 10–50 h was relatively slow. In the case of oxidized at 600 °C for 50 h, the specimens change appearance color to black after corrosion. Initially, the weight gain increased slowly, but after 20 h, the weight loss slowed substantially. After that, the specimens' weight loss is 0.54 mg/cm^2^ after 50 h, as shown in Fig. 13. The oxidized at 800 °C specimens were not changed their appearance color after corrosion. The weight change was low influenced where weight gain was 0.8 mg/cm^2^.

**Figure 14:**
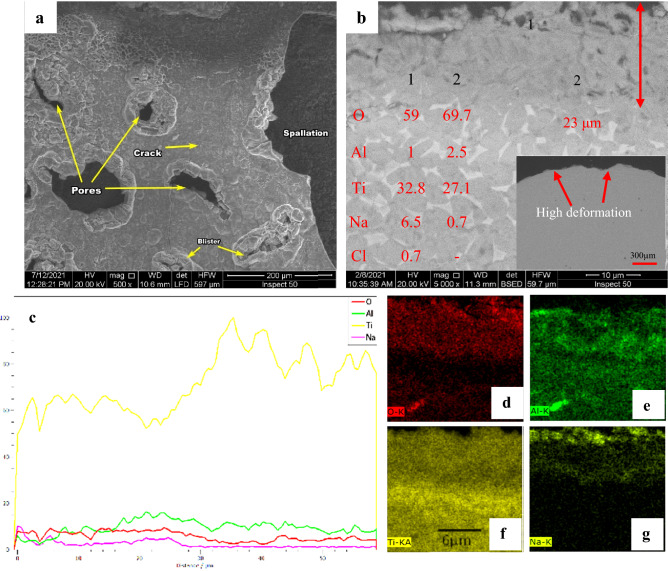
SEM and EDS for raw specimens after exposure to hot corrosion (**a**) surface morphology, (**b**) cross-section, (**c**) line element distribution, and (**d**–**g**) element map concentration.

Figure 14 shows SEM and EDS for raw specimens after exposure to hot corrosion. Raw surface morphology after hot corrosion has flaked off, high surface roughness, cracks, and the specimens have become an irregular outer shape. In addition, a very adverse consequence was the presence of holes, blisters, and poor adhesion between the base metal and produced scale. These significant changes of high porosity are detected on the surface of corrosion scales, as illustrated in Fig. 14a. The cross-sectional observation of the raw specimen, Fig. 14b, demonstrates that the average thickness of produced corrosion scales was 23 μm. Some cracking may occur in the substrate parallel to the layer. The presence of O, Ti, Al, and Na throughout the corrosion scale may be recognized by EDS elemental mapping, as seen in Fig. 14d-g. Na is identified nearly an extended scale, where a high concentration of Na layer in the top surface has an average thickness of 6.8 µm. The inner part of the scale is produced of Ti and O, and based on EDS analysis, and it is feasible to detect TiO_2_, the principal scale.

**Figure 15:**
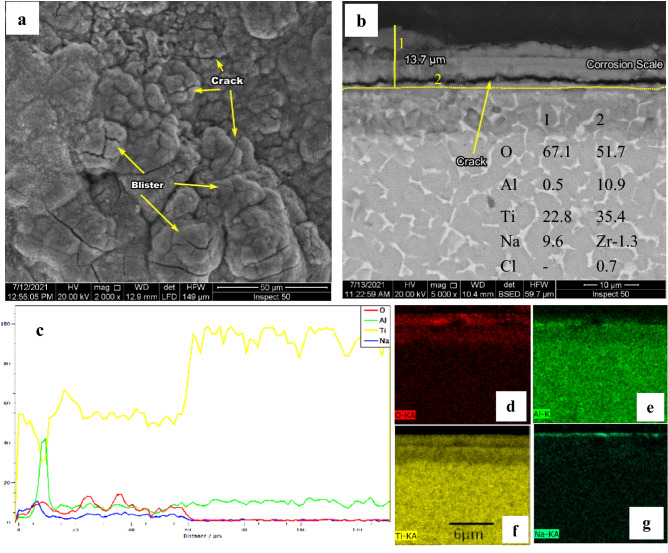
SEM and EDS analysis for oxide 600 °C after hot corrosion (**a**) surface morphology, (**b**) cross-section**,** (**c**) line element distribution, and (**d–g**) element map concentration.

Good corrosion resistance was seen in Fig. 15 for the oxidized specimen at 600 °C for 50 h. The surface morphology of specimens displays a crack, pores, and many blisters, as illustrated in Fig. 15a. SEM cross-section indicates crack and poor adhesion to the base metal in Fig. 15b. The scale thickness was 13.7 µm that larger than the layer was formed in oxidation. EDS elemental mapping shows that a thin Na produces the corrosion scale at the exterior portion with a thickness of 2.8 µm, a scale larger formed from the Ti and O layer.

**Figure 16:**
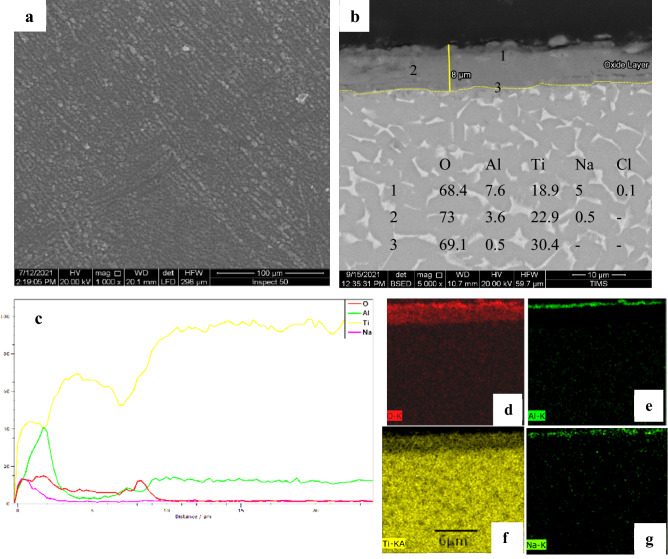
SEM and EDS analysis for oxide 800 °C after hot corrosion (**a**) surface morphology, (**b**) cross-section**,** (**c**) line element distribution, and (**d–g**) element map concentration.

Figure 16 shows SEM and EDS analysis for oxide 800 °C after hot corrosion. The best resistance against corrosion was seen in the form of the oxidized specimen at 800 °C. The surface morphology of the specimen was free from the crack, pores, and blisters, as illustrated in Fig. 16a. As illustrated in Fig. 16b, SEM pictures of cross-sections demonstrate that the protective oxide layer is regular and adheres to the substrate with an average thickness of 7.4 µm, smaller than formed in oxidation. According to EDS elemental mapping, the corrosion scale consists of a thin Na layer at the outer section of 1.2 µm, the thickness of the Al layer is 1.3 µm, and the main layer composition of O and Ti.

**Figure 17:**
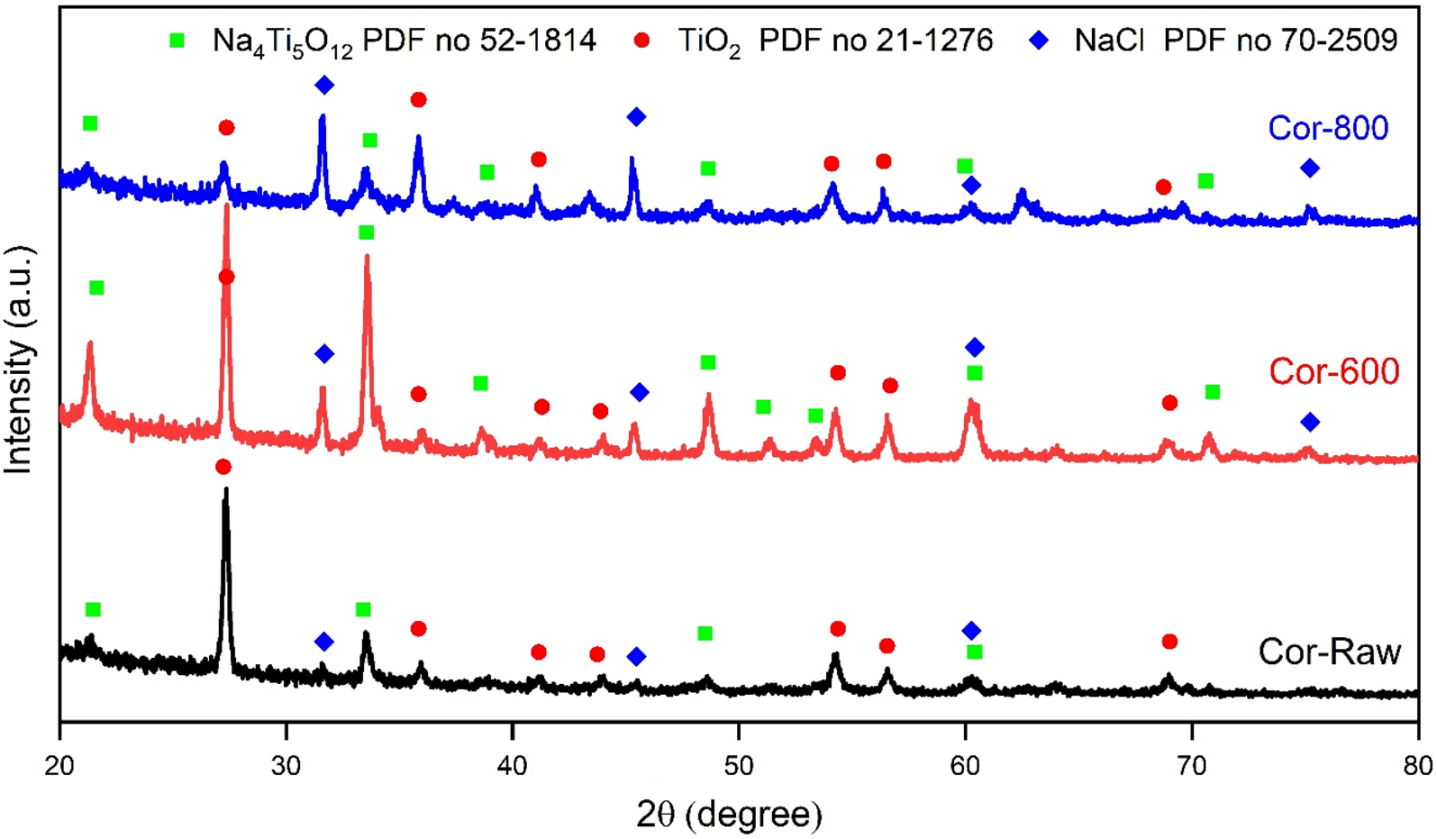
XRD patterns of raw and oxidized (600 and 800 °C/50 h) specimens after hot corrosion at 600 °C for 50 h.

Figure 17 exhibits XRD patterns of raw and oxidized (600 and 800 °C/50) specimens following hot corrosion at 600 °C for 50 h. TiO_2_ (PDF number 21-1276) and Na_4_Ti_5_O_12_ (PDF number 520-1814) are the primary corrosion products, except for tiny quantities of NaCl (PDF number 70-2509). The compositions of products varied when solid NaCl was put on the specimens, as illustrated in Fig. 17. The findings of this investigation were compatible with previous work^16,45^. Sn oxide layers are missing from the exterior corrosion scale. Some chlorides might be attributed to exceptionally poor stability as Ti and Al chlorides. Several researchers have explored the corrosion mechanism in Ti alloys with NaCl^46–49^. An active corrosion process is considered responsible for reducing corrosion protection in the presence of NaCl deposits. Some information about NaCl melts point at 800 °C and vaporizes at 1515 °C, the harmless reaction between Ti and NaCl above 400 °C^45,46^.

**Figure 18:**
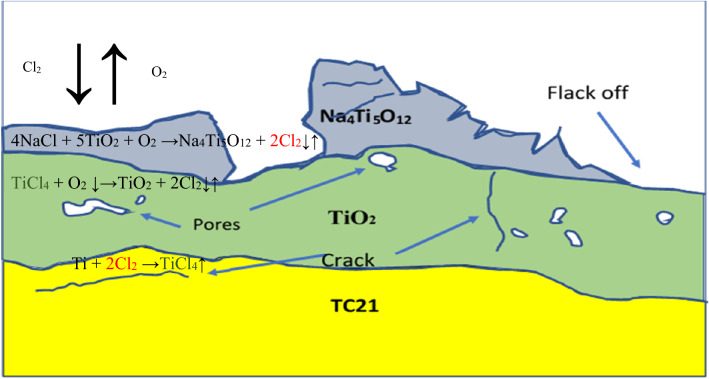
Schematic diagram was the corrosive layer formation under the raw material's hot corrosion condition.

Spalling and cracking were formed during the high-pressure level of gaseous chlorine in oxide scales. Table 3 standard Gibbs free energy changes reactions at 600 °C were represented as negative; thus, the reactions are thermodynamically spontaneous. Figure 18 represents a schematic diagram for forming the corrosive layer under the hot corrosion condition of raw material. At the surface reaction1 starts between TiO_2_, NaCl, and O_2_ to form a mixed Na_4_Ti_5_O_12_ and Cl_2_, which Na_4_Ti_5_O_12_ accumulated on the surface. Chlorine escapes part in the gaseous atmosphere, but some can diffuse through the crack and pores in the oxide layer toward the metal/oxide interface and react with metallic Ti to form TiCl_4_, as presented in reaction 2. The reaction 3 volatile TiCl_4_ reacts with O_2_ where TiCl_4_ transfer through cracks and pores in the corrosion layer to partial pressure P(O_2_) increase to induce reaction. This reaction released porous non-protective TiO_2_ and volatile Cl_2_, resulting in self-sustaining corrosion reactions on the metal/oxide interface. The TiCl_4_ released during the reaction (3) can be diluted in the gaseous atmosphere and react with metallic O_2_ in order to form a TiO_2_ but deposited at the surface as schematic in Fig. 18. Although, according to Ciszak C et al.^47^, many oxides can be observed according to theoretical calculations, in practice, TiO_2_, ZrO_2_, and Al_2_O_3_ have been observed, but only TiO_2_ was observed in this study.

**Table 3:** Reactions between NaCl and Ti with standard Gibbs free energy at 600 °C ^49^.

No	Reaction	ΔG° kJ/mol
1	Ti + O_2_ → TiO_2_	− 785
2	4NaCl + 5TiO_2_ + O_2_ → Na_4_Ti_5_O_12_ + 2Cl_2_	− 194
3	4NaCl + 6TiO_2_ → Na_4_Ti_5_O_12_ + TiCl_4_	− 65
4	TiCl_4_ + O_2_ → TiO_2_ + 2Cl_2_	− 129
5	Ti + Cl_2_ → TiCl_2_	− 374
6	Ti + 2Cl_2_ → TiCl_4_	− 656
7	TiCl_2_ + O_2_ → TiO_2_ + Cl_2_	− 411

At 600 °C for 50 h, NaCl catastrophe effect was deposited to base metal alloy; the weight loss was 6.4 mg/cm^2^. In the case of oxide at 600 °C, tiny increases weight gain duration first after 20 h, then gradually decreases to − 0.54 mg/cm^2^ of the initial weight after 50 h. high corrosion resistance appears oxide at 800, where the weight gain was 0.8 mg/ cm^2^ after 50 h. Due to the very thin Al layer, the oxide layer at 600 °C is not composed of protected Al_2_O_3_. At 600 °C, during 20 h, the weight gain of the specimen due to corrosion resistance by protective TiO_2_ is not consuming. At up to 20 h duration, TiO_2_ destroys the corrosion layer formed of discontinuous non-protective and cracks products on the coating. Coatings can separate due to corrosives diffusing rapidly inward through the porous channels. Hence, even though a continuous Al_2_O_3_ layer formed on oxide at 800 °C, the layer was protected from corrosion. Actually, the consumption of Al_2_O_3_ by the reactions with NaCl is very slow at a temperature of 600 °C. However, the decrease in the amount of external O_2_ reaching to base metal so become the difficult formation of the protective oxide layer in case of corrosion. The increased NaCl corrosion duration led to an increased consumption rate of Al_2_O_3_. The corrosion reactions reduce in the presence of protective and continuous Al_2_O_3_^48^.

## Conclusions

The effect of thermal oxidation oxide layers on hardness and wear rate on TC21 Ti-alloy was examined in this research. In addition, the NaCl-induced hot corrosion behavior of raw and oxidized specimens was examined. As the importance of this study, the following conclusions may be reached.

1. The average thickness of the oxide layer increased with oxidation temperature, where oxidized specimens at 600, 700, 800, and 900 were 0.47, 3.8, 9.7, and 5.1 µm, respectively. The oxide layer phases were mainly the TiO_2_ (rutile) and a small Al_2_O_3_.
2. The thermal oxidation temperature considerably improved the hardness and wear rate of TC21 alloy. The temperature has been revealed to more substantially affect oxide formation than time.
3. The most excellent surface hardness observed for the oxidized layer at 900 °C (1000 ± 150 HV_0.05_) was threefold higher than the hardness of raw specimens (364 ± 15 HV_0.05_).
4. The wear rate was improved with increasing oxidation temperature and duration, where the wear rate for the oxidized layer for specimens treated at 800 °C was 2.3 × 10^–5^ g/sec, which is four times less than that of raw specimens (10.8 × 10^–5^ g/sec).
5. The presence of solid NaCl deposits on the TC21 alloy surface is highly damaging. The weight loss after hot corrosion at 600 for 5 cycles was 6.4 mg/cm^2^ for raw metal compared to 0.54 mg/cm^2^ for the specimen oxidized at 600 °C.
6. The weight gain of the oxidized specimen at 800 °C was 0.8 mg/cm^2^ after hot corrosion at 600 °C for 5 cycles. The specimen displayed the most considerable regular layer and excellent results in corrosion resistance.

## Data Availability

All data generated or analyzed during this study are included in this published article [and its supplementary information files.

## References

[1] Kolli RP, Devaraj A (2018). A review of metastable beta titanium alloys. Metals (Basel)..

[2] Liu Z, He B, Lyu T, Zou Y (2021). A review on additive manufacturing of titanium alloys for aerospace applications: Directed energy deposition and beyond Ti-6Al-4V. JOM.

[3] Elshaer, R. N. Effect of initial α-phase morphology on microstructure, mechanical properties, and work-hardening instability during heat treatment of TC21 Ti-Alloy. *Metallogr. Microstruct. Anal.* 1–16 (2022).

[4] Wang S, Liao Z, Liu Y, Liu W (2015). Influence of thermal oxidation duration on the microstructure and fretting wear behavior of Ti6Al4V alloy. Mater. Chem. Phys..

[5] Tong ZP (2019). Effect of laser shock peening on wear behaviors of TC11 alloy at elevated temperature. Opt. Laser Technol..

[6] Aniołek K (2017). The influence of thermal oxidation parameters on the growth of oxide layers on titanium. Vacuum.

[7] Bailey R, Sun Y (2013). Unlubricated sliding friction and wear characteristics of thermally oxidized commercially pure titanium. Wear.

[8] Aniołek, K., Barylski, A., Kupka, M. and Tylka, J. The influence of thermal oxidation parameters on structural, friction, and wear characteristics of oxide layers produced on the surface of Ti–6Al–7Nb Alloy. *J. Tribol.* **141**, (2019).

[9] Sun Q, Hu T, Fan H, Zhang Y, Hu L (2016). Thermal oxidation behavior and tribological properties of textured TC4 surface: Influence of thermal oxidation temperature and time. Tribol. Int..

[10] Abdo HS, Sherif E-SM, El-Serehy HA (2020). Manufacturing of ti-6% al and ti-6% al-4% v alloys and their corrosion in sodium chloride solutions. Crystals.

[11] Aniołek K, Barylski A, Kupka M, Leszek I (2020). The tensile properties, scratch behaviors and sliding wear of oxide scale formed on titanium Grade 2. Materials (Basel)..

[12] Gurrappa I (2003). Mechanism of degradation of titanium alloy IMI 834 and its protection under hot corrosion conditions. Oxid. Met..

[13] Gao PF, Lei ZN, Wang XX, Zhan M (2019). Deformation in fatigue crack tip plastic zone and its role in crack propagation of titanium alloy with tri-modal microstructure. Mater. Sci. Eng. A.

[14] Pambudi, M. J., Basuki, E. A. and Prajitno, D. H. Improving hot corrosion resistance of two phases intermetallic alloy α2-Ti3Al/γ-TiAl with enamel coating. in *AIP Conference Proceedings* vol. 1805 70003 (AIP Publishing LLC, 2017).

[15] Li R (2020). A new insight into the NaCl-induced hot corrosion mechanism of TiN coatings at 500 °C. Corros. Sci..

[16] Dai J, Zhu J, Chen C, Weng F (2016). High temperature oxidation behavior and research status of modifications on improving high temperature oxidation resistance of titanium alloys and titanium aluminides: A review. J. Alloys Compd..

[17] Kitashima T, Hara T, Yang Y, Hara Y (2018). Oxidation–nitridation-induced recrystallization in a near-α titanium alloy. Mater. Des..

[18] Casadebaigt A, Hugues J, Monceau D (2020). High temperature oxidation and embrittlement at 500–600 °C of Ti-6Al-4V alloy fabricated by laser and electron beam melting. Corros. Sci..

[19] Dai J (2021). High temperature oxidation and hot corrosion behaviors of Ti2AlNb alloy at 923 K and 1023 K. Corr. Sci.

[20] Elshaer RN, Ibrahim KM (2020). Effect of cold deformation and heat treatment on microstructure and mechanical properties of TC21 Ti alloy. Trans. Nonferrous Met. Soc. China.

[21] Elshaer RN, El-Deeb MS, Mohamed SS, Ibrahim KM (2021). Effect of strain hardeningand aging processes on microstructure evolution, tensile and fatigue properties of cast Ti-6Al-2Sn-2Zr–2Mo-1.5Cr-2Nb-0.1Si Alloy. Int. J. Met..

[22] Cai J (2020). Hot corrosion behavior of arc ion plating NiCoCrAlYSiHf coating via high-current pulsed electron beam. Oxid. Met..

[23] Elshazli AM, Elshaer RN, Hussein AHA, Al-Sayed SR (2021). Laser surface modification of TC21 (α/β) titanium alloy using a direct energy deposition (DED) process. Micromachines.

[24] Elshaer RN, Elshazli AM, Hussein AHA, Al-Sayed SR (2022). Impact of laser process parameters in direct energy deposition on microstructure, layer characteristics, and microhardness of TC21 alloy. Int. J. Adv. Manuf. Technol..

[25] Zhang L (2020). Oxidation resistance of plasma-sprayed double-layered LC/YSZ coatings with different thickness ratios at high temperatures. Oxid. Met..

[26] Ding Z-Y (2019). Influence of Al2O3 addition in NaAlO2 electrolyte on microstructure and high-temperature properties of plasma electrolytic oxidation ceramic coatings on Ti2AlNb alloy. Surf. Coatings Technol..

[27] Ye F, Zhao L, Mu C, Zhao H (2017). Influence of yttrium addition on reactive sputtered W-Y–N coatings. Surf. Eng..

[28] Abdo HS, Abdus Samad U, Mohammed JA, Ragab SA, Seikh AH (2021). Mitigating corrosion effects of Ti–48Al–2Cr–2Nb alloy fabricated via electron beam melting (EBM) technique by regulating the immersion conditions. Crystals.

[29] Guo, X., Zhang, J., Chen, B. and Li, J. Effects of triple heat treatment on microstructure and the dry sliding wear of TC21 titanium alloy. in *IOP Conference Series: Materials Science and Engineering* vol. 768 22067 (IOP Publishing, 2020).

[30] Wang S, Liao Z, Liu Y, Liu W (2014). Influence of thermal oxidation temperature on the microstructural and tribological behavior of Ti6Al4V alloy. Surf. Coatings Technol..

[31] Guleryuz H, Cimenoglu H (2009). Oxidation of Ti-6Al–4V alloy. J. Alloys Compd..

[32] Kumar S, Narayanan TSNS, Raman SGS, Seshadri SK (2010). Thermal oxidation of Ti6Al4V alloy: Microstructural and electrochemical characterization. Mater. Chem. Phys..

[33] McReynolds KS, Tamirisakandala S (2011). A study on alpha-case depth in Ti–6Al–2Sn–4Zr–2Mo. Metall. Mater. Trans. A.

[34] Kumar A, Kushwaha MK, Israr M, Kumar R (2020). Evaluation of mechanical properties of titanium alloy after thermal oxidation process. Trans. Indian Inst. Met..

[35] Aniołek K (2020). Structure and properties of titanium and the Ti–6Al–7Nb alloy after isothermal oxidation. Surf. Eng..

[36] Jia Z (2015). The color changes and tensile properties of oxidized Ti–6A1–2Mo–1.5 Cr–2Zr–2Sn–2Nb alloy. J. Alloys Compd..

[37] Wang X, Zhao Y, Hagiwara M, Hou H, Suzuki T (2010). Kinetics of dehydrogenation in Ti600, TC21 and Ti40 alloys. J. Alloys Compd..

[38] Sarma J, Kumar R, Sahoo AK, Panda A (2020). Enhancement of material properties of titanium alloys through heat treatment process: A brief review. Mater. Today Proc..

[39] Du HL, Datta PK, Lewis DB, Burnell-Gray JS (1994). Air oxidation behaviour of Ti–6Al–4V alloy between 650 and 850°. Corros. Sci..

[40] Aniołek K (2021). mechanical properties, corrosion resistance and bioactivity of oxide layers formed by isothermal oxidation of Ti–6Al–7Nb alloy. Coatings.

[41] Dalili N (2010). Improving the wear resistance of Ti–6Al–4V/TiC composites through thermal oxidation (TO). Wear.

[42] Aniołek K, Kupka M (2019). Mechanical, tribological and adhesive properties of oxide layers obtained on the surface of the Ti–6Al–7Nb alloy in the thermal oxidation process. Wear.

[43] Latief FH, Sherif E-SM, Wismogroho AS, Widayatno WB, Abdo HS (2020). The cyclic oxidation and hardness characteristics of thermally exposed titanium prepared by inductive sintering-assisted powder metallurgy. Crystals.

[44] Guleryuz H, Cimenoglu H (2005). Surface modification of a Ti–6Al–4V alloy by thermal oxidation. Surf. Coatings Technol..

[45] Ciszak C, Popa I, Brossard J-M, Monceau D, Chevalier S (2016). NaCl induced corrosion of Ti–6Al–4V alloy at high temperature. Corros. Sci..

[46] Fan L (2019). Effect of streaming water vapor on the corrosion behavior of Ti60 alloy under a solid NaCl deposit in water vapor at 600 °C. Corros. Sci..

[47] Ciszak C (2020). Degradation mechanism of Ti–6Al–2Sn–4Zr–2Mo–Si alloy exposed to solid NaCl deposit at high temperature. Corros. Sci..

[48] Xiong Y, Zhu S, Wang F (2008). Synergistic corrosion behavior of coated Ti60 alloys with NaCl deposit in moist air at elevated temperature. Corros. Sci..

[49] Fan L (2016). Corrosion behavior of Ti60 alloy under a solid NaCl deposit in wet oxygen flow at 600 °C. Sci. Rep..

